# Physical and Mental Health Related Quality of Life and Their Influencing Factors on Sexual Minority Women in Korea

**DOI:** 10.3390/ijerph18042115

**Published:** 2021-02-22

**Authors:** Ssirai Kim, Smi Choi-Kwon

**Affiliations:** 1 College of Nursing, Seoul National University, Seoul 03080, Korea; ssirai@snu.ac.kr; 2 Department of Healthcare Administration, Seoul National University Hospital, Seoul 03080, Korea; 3 The Research Institute of Nursing Science, Seoul National University, Seoul 03080, Korea

**Keywords:** mental health, physical health, sexual minority women, health-related quality of life, Korea

## Abstract

Korean sexual minority women (SMW) often experience discrimination, but their health-related quality of life (HRQoL) remains to be investigated. Therefore, we aimed to assess the levels of mental and physical HRQoL of Korean SMW and their influencing factors using data from the Korean Sexual Minority Women’s Health Study (2017) in a cross-sectional study, which included lesbian and bisexual females (*N* = 736; age ≥19 years). The HRQoL was measured using SF-36v2^®^; moreover, separate multiple linear regression analyses were conducted to identify the factors influencing mental and physical HRQoL. The physical and mental HRQoL scores were average (52.38 ± 7.65) and low (38.33 ± 12.64), respectively. Significant factors influencing the physical HRQoL were bisexuality, minority stress, perceived social support, and physical activity. The same factors—apart from physical activity—were associated with mental HRQoL. Therefore, to improve the HRQoL of SMW, it is necessary to lower their minority stress and increase social support. Moreover, special attention is needed regarding bisexual women in Korea.

## 1. Introduction

Health inequities among sexual minority women (SMW) are well-established. Compared with heterosexual women, SMW experience worse mental health outcomes [1,2,3,4,5], including anxiety and depression, and have poor physical health [1,6,7], including high-risk health-related lifestyle factors [8,9] such as smoking and alcohol abuse. Furthermore, SMW’s health-related quality of life (HRQoL) is lower than their heterosexual counterparts [10,11,12,13,14,15].

The low health level of SMW may be due to minority stress, the unique and additive stress experienced by people with minority identities [5,16]. According to Minority Stress Theory [5,17], stress from stigmatized identity status or stigmatizing societal structure negatively affects health. Previous studies [3,5] reported that distal stress, such as violence or victimization against sexual identity, was related to the poor mental health of sexual minority people. Proximal stress-vigilance or internalized homophobia also related to an individual’s perceptions and predicted adverse health outcomes [3,4]. On the other hand, in the Psychological Mediation Framework, stress could result in maladaptive coping and emotion regulation that increases the risk of poor mental health [16,18,19]. Moreover, when exposed to stressors, fear of disclosing identities prevents sexual minority people from feeling sufficiently supported [16]. However, sexual minorities are not only passive victims of stress; their identity can also be a protective factor [17]. One of the most positive effects on the health of SMW is social support [1,2,20]. Community connectivity was also addressed as another positive factor affecting mental health [20]. Furthermore, factors that affect mental health also appear to affect physical health [1,6,21]. Specifically, high minority stress, low physical activity, and various health-related lifestyle factors are related to low physical HRQoL [21]. Another important point when describing the health of SMW is the difference between inner groups. In previous studies, bisexual women reported poorer health than lesbians [1,3,15,22,23]. However, most of these findings were reported in Western countries [11,12,13,14,15,21].

The expression of hatred toward sexual minorities has emerged as an important social issue in Korea [24,25]. Christianity, the religion that more than a third of Koreans belong to, shows strong opposition to sexual minorities, more so than other religions [26]. In addition, a traditional family system that values heterosexuality, and deep-rooted Confucian virtues, are hallmarks of the anti-LGBT atmosphere in Korea [25,27,28,29]. These societal concerns pose a threat to the health of sexual minorities. Previous studies showed a higher prevalence of depressive symptoms and suicidal ideation among sexual minorities than the general population in Korea [30,31]. Depressive symptoms were related to discrimination against sexual orientation or lower community connectedness [31]. On the other hand, family support was a significant factor that impacted life satisfaction among Korean sexual minorities [32].

A previous study highlighted the need to research the health of lesbians and bisexual women since they are relatively ignored in Korean sexual minority health studies [30]. Past studies have explored the quality of life or life satisfaction in Korean sexual minorities, but participants were limited to gay men or lesbians only [32,33]. Few studies examined the comprehensive health status of Korean SMW including bisexuals.

Therefore, we attempted to examine the level of physical and mental HRQoL in Korean SMW and identify the factors associated with each HRQoL. Our main hypotheses were as follows: (1) SMW in Korea may have a low level of HRQoL, (2) high minority stress level lowers the physical and mental HRQoL of SMW in Korea, (3) high perceived social support positively affect physical and mental HRQoL, and (4) Korean bisexual women have lower HRQoL than lesbians do.

## 2. Materials and Methods

### 2.1. Study Design and Participants

This cross-sectional study used data from the “Korean Sexual Minority Women’s Health Study”, which was conducted to examine Korean SMW’s HRQoL and health behaviors including health service utilization. The data were collected via an online survey form using SurveyMonkey, a survey hosting site, from September to November 2017. The participants of the study included Korean women who were 19 years or older, lesbian, bisexual, or belonged to other sexual minorities. Gender minorities were excluded from the study. We recruited the participants through social media, LGBT organizations’ newsletters, or online LGBT communities including those targeted towards SMW over 30 years old. Participants were informed about the study’s purpose and design on the study webpage. Among the 1256 respondents who answered questions in the survey, only 801 fully completed the survey. Further, we excluded those who chose “asexual”, “uncertain”, or “other” for the sexual identity question (*N* = 65). In total, 736 SMW were selected.

### 2.2. Study Variables

#### 2.2.1. Sociodemographic Variables and Sexual Identity

Sociodemographic characteristics included age (groups: 19–29, 30–39, and 40–51), city of residence (capital city—Seoul, or other cities), education level (high school or pre-college and graduate), employment status (student, employed, or unemployed), and annual family income level. Annual family income was divided into <USD 50,000 and ≥USD 50,000 based on the average income per Korean household at the time of survey—1 USD equaled approximately 1000 KRW [34]. Participants who described themselves as lesbian, bisexual, or pansexual were included in this study. We categorized them into two groups: lesbian and bisexual (including pansexuals).

#### 2.2.2. Health-Related Lifestyle Factors

We examined the following health-related lifestyle factors: smoking (non-smokers and smokers, who answered “Yes” to the question “Are you currently a smoker”), high-risk drinking (two drinks at a time twice weekly), and physical activity [35]. Physical activity was measured using the Korean version of the International Physical Activity Questionnaire (IPAQ) [36], and then categorized as low, moderate, and high activity.

#### 2.2.3. Minority Stress

To measure day-to-day minority stress, we used the Daily Heterosexist Experience Questionnaire (DHEQ) developed by Balsam et al. [37] with permission from the original author. The measure was translated into Korean through reverse translation. Out of the original nine subgroups (50 items), we used seven—vigilance, discrimination and harassment, gender expression, victimization, family of origin, vicarious trauma, and isolation. We excluded parenting and HIV subscales in the current study because of the legal and social context. Under Korean law, same-sex marriages are not allowed, and single parent adoptions are rarely implemented. Moreover, the prevalence of HIV among Korean women is much lower than among men [38]. Participants responded to “How much has this problem distressed or bothered you during the past 12 months?” by choosing from the options ranging from 0 (did not happen/not applicable to me) to 5 (it happened, and I was extremely bothered) on a six-point Likert scale. Responses were later recoded so that both “0” and “1” represented “1” (did not bother me). The rest of the responses remained the same. Subsequently, we computed a mean score from the responses to all items, reflecting the mean level of distress a participant feels related to these experiences. Higher scores indicated higher stress related to experiencing, and being bothered by, instances of heterosexism. We used 35 items for this study after factor analysis. At the time of development, Cronbach’s α of each subgroup ranged from 0.72 to 0.87 [37], and the overall Cronbach’s α was 0.855 in this study.

#### 2.2.4. Perceived Social Support

The Multidimensional Scale of Perceived Social Support (MSPSS)—available for free from http://gzimet.wixsite.com/mspss (accessed on 30 May 2017)—was used to measure the perceived social support from three sources: significant other, family, and friends [39]. The scale consists of 12 items with response options presented on a seven-point Likert-type scale, ranging from 1 (very strongly disagree) to 7 (very strongly agree). Items included statements such as “There is a special person who is around when I am in need,” and “My family really tries to help me.” The total MSPSS score was calculated by adding the scores of all 12 items and dividing the total score by 12. Each domain score was obtained in the same way by using 4 as the divisor. Higher MSPSS scores reflected higher perceived social support. Cronbach’s α of the MSPSS was 0.91 at the time of development and 0.893 in this study.

#### 2.2.5. Physical and Mental Health-Related Quality of Life

To measure HRQoL, we used a standardized SF-36v2^®^ Korean version by Optum (License no.: QM041038), consisting of 36 items and eight domains. The domains were physical functioning, bodily pain, role physical, and general health, all included in the physical component summary (PCS) calculation; and mental health, social functioning, role emotional, and vitality, included in the calculation of the mental component summary (MCS). In our study, MCS indicates mental-health-related quality of life (Mental HRQoL) and PCS indicates physical-health-related quality of life (Physical HRQoL)

By using *QualityMetric* software (QualityMetric Incorporated, Lincoln, RI, USA), we converted the data from the SF-36v2^®^ to a numerical score using norm-based scoring, normalized so that each domain’s mean score was 50 (*STD* ± 10) for the general population. Mean scores below 47 and above 53 indicated poorer and better health status, respectively, than the normal range [40].

### 2.3. Statistical Analysis

The number of samples for this study was set based on a significance level of 0.05, a medium effect size of 0.15, a power of 0.95, and a total of 12 variables required for regression analysis. As a result of the analysis using G*power 3.1 software [41], the minimum number of samples was 184, indicating that this study satisfies the appropriate number of samples. We used complete case analysis to deal with missing data, and only those who reported complete data were included in the analysis. We also checked outliers for entry errors and used all 736 completed data for final analysis.

First, to examine the HRQoL and explanatory variables of Korean SMW, we used descriptive statistics and conducted bivariate analysis. Age was calculated as median and inter-quartile ranges. Categorical variables are presented as counts and proportions, and continuous variables are presented as mean and standardized deviation (*SD*). We performed factor analysis and calculated Cronbach’s α. The participants’ physical and mental HRQoL were compared according to their characteristics, using t-test or ANOVA. Moreover, post hoc *F* tests were conducted to detect potential differences among more than three groups. The correlation between the continuous variables and HRQoL was explored through Pearson’s correlation analysis. Second, separate multiple linear regression analyses were performed to determine factors influencing physical and mental HRQoL. To check whether the assumptions of regression analysis were satisfied, tolerance, variation inflation factor, and Durbin-Watson were calculated for each model. In the first model (Model 1), we examined the association between sexual identity and HRQoL variables, controlling for sociodemographic variables. We added smoking, high-risk drinking, and physical activity level in the second model (Model 2). Minority stress was included in Model 3. In the last model (Model 4), perceived social support was added. All statistical analyses were conducted using IBM SPSS Statistics for Windows, Version 25.0 (IBM Corp, Armonk, NY, USA), except the scores of HRQoL, which were initially calculated using QualityMetric (QualityMetric Incorporated, Lincoln, RI, USA) software. *p*-values were based on a two-sided significance level of 0.05.

### 2.4. Ethics Declarations

We obtained informed consent from the study participants via an online survey form. As a reward for their participation, we provided two options: an online coupon equivalent to $5 if subjects left their contact details, or a $5 donation to LGBT organizations if they wanted to keep their identity private. The Korean Sexual Minority Women’s Health Study was approved by the institutional review board of the Seoul National University (IRB No. 1707/002-005).

## 3. Results

### 3.1. Health-Related Quality of Life by Participants’ Characteristics

The median age of the Korean SMW participants was 25, and 50.8% were lesbian. Of the total participant group, 23% were current smokers and 11% showed high-risk drinking. Furthermore, 75% had been engaged in moderate to high physical activity, and the mean physical HRQoL of all 736 participants was 52.38 ± 7.65. Interestingly, the physical HRQoL of the participants showed significant differences in terms of education level (*p* = 0.017), employment status (*p* = 0.012), sexual identity (*p* = 0.026), smoking (*p* = 0.031), and physical activity (*p* < 0.0001), Table 1.

**Table 1:** Health-Related Quality of Life by Participants’ Characteristics.

	Total		Health-Related Quality of Life							
			Physical (PCS)				Mental (MCS)			
	N or M	% or SD	M	SD	t/F or r (Scheffe)	*p*-Value	M	SD	t/F or r (Scheffe)	*p*-Value
Total	736	100	52.38	7.65			38.33	12.64		
Age group										
19–29(a)	559	76.0	52.59	7.81			37.44	12.56	7.877	<0.0001
30-39(b)	146	19.8	51.59	6.94	0.998	0.369	40.28	12.96	(a < c)	
40-51(c)	31	4.2	52.27	7.91			45.22	9.09		
Median (Q1–Q3)	25 (21–29)									
City										
Capital city	344	46.7	52.29	7.36	−0.275	0.783	38.55	12.39	0.445	0.656
Other	392	53.3	52.45	7.90			38.14	12.86		
Education										
High school or under	70	9.5	50.30	7.41	2.396	0.017	35.71	13.80	1.684	0.096
Above college	666	90.5	52.59	7.64			38.61	12.49		
Annual family income										
<USD 50,000	525	71.3	52.04	7.66	−1.858	0.064	37.66	12.93	−2.372	0.018
≥USD 50,000	211	28.7	53.20	7.57			40.00	11.74		
Employment										
Student(a)	349	47.4	53.14	7.81	4.413		36.93	12.81	14.268	<0.0001
Employed(b)	325	44.2	51.44	7.20	(b < a)	0.012	40.83	11.52	(a,c < b)	
Unemployed(c)	62	8.4	53.01	8.54			33.14	14.51		
Sexual Identity										
Lesbian	374	50.8	52.99	7.68	2.233	0.026	39.90	12.52	3.436	0.001
Bisexual	362	49.2	51.74	7.57			36.72	12.56		
Smoking										
Yes	173	23.51	51.28	7.79	2.161	0.031	37.54	12.81	0.947	0.344
No	563	76.49	52.71	7.58			38.58	12.58		
High -risk drinking										
Yes	85	11.54	51.452	8.0581	1.185	0.236	39.281	13.9093	−0.736	0.462
No	651	88.46	52.496	7.5897			38.208	12.4657		
Physical Activity										
High (a)	249	33.83	53.17	7.06	8.552		39.34	12.22		
Moderate (b)	305	41.44	52.92	7.60	(c < a,b)	<0.0001	38.36	12.32	1.953	0.143
Low (c)	182	24.73	50.37	8.17			36.91	13.60		
Minority Stress	2.14	0.48			−0.151	<0.0001			−0.238	<0.0001
Perceived Social Support	4.89	1.11			0.124	0.001			0.428	<0.0001

PCS: Physical Component Summary, MCS: Mental Component Summary; N: Number; M: Mean; SD: Standard Deviation.

The mean of the mental HRQoL of all 736 participants was 38.33 ± 12.64. The mental HRQoL of the participating SMWs showed significant differences in terms of age group (*p* < 0.0001), annual family income (*p* = 0.018), employment status (*p* < 0.0001), and sexual identity (*p* = 0.001).

The mean minority stress (2.14 ± 0.48) was negatively correlated with both physical (*p* < 0.0001) and mental (*p* < 0.0001) HRQoL. In contrast, the mean perceived social support (4.89 ± 1.11) showed a positive correlation with both physical (*p* = 0.001) and mental (*p* < 0.0001) HRQoL.

### 3.2. Subdomains of Minority Stress and Perceived Social Support

We further analyzed the subdomains of minority stress and perceived social support to determine the characteristics of those variables. Among the minority stress subdomains, stress due to vicarious trauma (3.77 ± 0.91) was the highest, followed by stress from vigilance (3.04 ± 1.14); moreover, the lowest stress level was from victimization (1.03 ± 0.23). Among the subdomains of perceived social support, the support from significant others (5.54 ± 1.39) was the highest, followed by friends (5.33 ± 1.38). The support from family was the lowest (3.81 ± 1.59), Table 2.

**Table 2:** Subdomains of Minority Stress and Perceived Social Support (N = 736).

	Range	Mean	SD
**Minority Stress**	1~5	2.14	0.48
Vigilance	1~5	3.04	1.14
Discrimination & Harassment	1~5	1.53	0.76
Gender expression	1~5	1.29	0.56
Victimization	1~5	1.03	0.23
Family of origin	1~5	1.40	0.68
Vicarious trauma	1~5	3.77	0.91
Isolation	1~5	2.21	0.94
**Perceived Social Support**	1~7	4.89	1.11
Significant other	1~7	5.54	1.39
Family	1~7	3.81	1.59
Friends	1~7	5.33	1.38

### 3.3. Predictors of Physical Health-Related Quality of Life

The factors affecting the physical HRQoL of our subjects are presented in Table 3. There is no multi-collinearity problem between the independent variables. In Model 1, employment status and sexual identity had a statistically significant influence on physical HRQoL, explaining the physical HRQoL variance of 2.1% (*p* = 0.003). In Model 2, employment status, sexual identity, and physical activity were statistically significant factors influencing physical HRQoL. The explanatory power of Model 2 increased to 4.2% (*p* < 0.0001). In Model 3, employment status, sexual identity, physical activity, and minority stress were statistically significant factors influencing physical HRQoL. The explanatory power of Model 3 increased to 5.5% (*p* < 0.0001). In the final model (Model 4), employment status (*p* = 0.03), sexual identity (*p* < 0.001), physical activity (*p* < 0.001), minority stress (*p* = 0.003), and perceived social support (*p* = 0.020) were significant factors influencing physical HRQoL. These results confirmed this study’s hypothesis. The explanatory power of Model 4 was 6.1% (*p* < 0.0001).

### 3.4. Predictors of Mental-Health-Related Quality of Life

The factors affecting the mental HRQoL of our participants are presented in Table 4. There is no multicollinearity problem between independent variables. In Model 1, age, education level, annual family income, employment status, and sexual identity had a statistically significant influence on mental HRQoL, explaining mental HRQoL variance of 5.3% (*p* < 0.0001). In Model 2, age, employment status, sexual identity, and physical activity were statistically significant factors influencing mental HRQoL. The explanatory power of Model 2 increased to 6.0% (*p* < 0.0001). In Model 3, annual family income, employment status, sexual identity, physical activity, and minority stress were statistically significant factors influencing mental HRQoL. The explanatory power of Model 3 increased to 11.3% (*p* < 0.0001). In the final model (Model 4), employment status (*p* < 0.0001), sexual identity (*p* < 0.0001), minority stress (*p* < 0.0001), and perceived social support (*p* < 0.0001) were significant factors impacting mental HRQoL. This result confirmed the study’s hypothesis. The explanatory power of Model 4 was 24.7% (*p* < 0.0001).

## 4. Discussion

To the best of our knowledge, this is the first study to examine HRQoL and its influencing factors among SMW in Korea. We found that the mental HRQoL of Korean SMW participants was low with a score level of 38.33 ± 12.64, whereas, the physical HRQoL score was 52.38 ± 7.65, which is in the average range. Interestingly, minority stress, perceived social support, and sexual identity affected both the physical and mental HRQoL of the SMW, but physical activity was related only to their physical HRQoL. Furthermore, their mental HRQoL was very low, to the point of clinically impaired functioning [40]. The mental HRQoL was even lower than that of other Korean groups measured with the same tool, including people with fatigue (45.4~47.8) or patients with liver diseases (46.2 ± 11.7) [42,43,44]. Our finding is similar to other studies that revealed a lower HRQoL among sexual minorities than the general population [10,11,12,22,45,46]. In terms of international comparison, the mental HRQoL score in this study was lower than that of the lesbians in some countries overseas (49.10 ± 10.10) or transgender (41.60 ± 12.76) groups [46,47].

The low mental HRQoL may, in part, be due to minority stress, which was confirmed as a risk factor during the regression analysis. These results were similar to previous studies [1,2,12,15,48]. However, our participants’ minority stress type was different: they rarely reported stress from events marked by direct discrimination (including being physically assaulted) due to sexual orientation. In contrast, some previous studies reported the experience of direct victimization [37,49]. Furthermore, we found that vicarious trauma in our subjects was high. In particular, the item analysis showed the highest level of stress from vicarious trauma, especially due to experiences such as “Hearing politicians say negative things about LGBT people” or “Hearing about LGBT people you know/do not know being treated unfairly” [37]. As this study was conducted in 2017, it could be assumed that the stress of Korean SMW was elevated when they encountered negative remarks about homosexuality during the Korean presidential election campaign [50]. Moreover, some previous studies pointed out the role of distal stress [3,49] when, for example, frequent exposure to negative media messages about sexual minorities were associated with greater psychological distress [2]. The results of our study showed that even if discrimination was not experienced physically, hearing that others were being discriminated against could impact mental health in the same way as experiencing it firsthand.

Furthermore, our subjects experienced high stress due to vigilance, which is related to proximal stress. In hiding their identities, Korean SMW experience increased stress. In previous studies, chronic experiences of discrimination and rejection were reported to increase vigilance [16]. Stress from stigma induced self-monitoring and vigilant concealing of one’s stigmatized identity. Korean SMW could have developed a coping mechanism to conceal their identity in general situations to avoid discrimination. In other words, poor mental health could have been caused by not only experiencing direct discrimination but also changing the coping and emotion-regulation process [16]. However, since our study did not assess the direct relationship among stressors, general psychological processes, and psychopathology, further research is needed in the future.

On the other hand, high social support was related to higher mental HRQoL among Korean SMW participants. Perceived social support not only positively affected mental HRQoL, but was the most influential factor. Similarly, in previous studies, the social support factor protected the mental health and quality of life of sexual minorities [13,51,52]. When we examined the sources of social support, Korean SMW received a high level of social support from significant others and friends. It seems that Korean SMW are actively forming positive relationships in the chosen relationship.

However, the level of social support in our study was somewhat lower than that found in previous studies [1,53]. In particular, we found that family social support was the lowest. These results have important implications for family-oriented Korean society. In previous Korean studies, family solidarity had a significant effect on the quality of life of sexual minorities, and family support was a significant factor in their life satisfaction [32,33]. Low family support could be the consequence of exposure to stigma-related stress, which may lead to concealment of self-identity to avoid rejection [16]. In this study, “I can talk about my problems with my family” showed the lowest result in item analysis. It was significantly lower than items like “My family really tries to help me”. This result may be indirectly related to the fear of revealing sexual orientation. However, future research is needed to see the relationship between stressors and lower supports. Moreover, in our study, groups with low family support often had low annual family income or education levels. The low socio-economic status (SES) itself may have hampered Korean SMW from receiving sufficient family support. Therefore, additional attention is needed to enhance support for Korean SMW who experience low family support.

In our study, bisexual identity also explained the low mental HRQoL of Korean SMW. Similar to what was found in other studies [1,3,15,22,23], bisexual women had poorer HRQoL than lesbians. There appear to be health disparities among Korean SMW: bisexuals may additionally experience bi-specific stress [54,55,56,57,58]. South Korea’s LGBT community is still growing and is characterized by a well-established majority of gay and lesbian organizations. In such an environment, bisexual women may feel increasingly isolated because they do not have access to communities that meet their needs and hence do not receive the required support [15,59].

Our results indicate that the physical HRQoL (52.38 ± 7.65) of Korean SMW was in the average or normal range [40]. This differs from previous studies where sexual minorities’ physical HRQoL was lower than that of the general population [13] and may be related to the younger age of participants in this study. Few severe disease-related or health status cases were found among the young people in their 20–30s in the Korean general population study [60]. In our findings, physical activity increased physical HRQoL. It could be assumed that our participants had good physical HRQoL because 70% of them reported moderate or high physical activity. Surprisingly, however, we found that their smoking rate was 23.5%—much higher than the 3.5–7.5% of the general Korean female population that smokes [35,61]. Smoking was not a significant variable that directly affected physical HRQoL, but the high smoking rate suggested that they may be more likely to develop cardiovascular disease later in life [62]. Considering that both stress and smoking are risk factors for cardiovascular disease, long-term studies focused on the physical health of Korean SMW are needed [1,6,8].

Our study has the following implications for the future establishment of public health strategies. First, our study shows that structural and national intervention is required to maintain and improve the health of Korean SMW. Destigmatizing policies such as anti-discriminatory laws should be actively implemented to reduce exposure of SMW to minority stress. In addition, the government should be able to accurately identify the health status of SMW by including variables related to sexual orientation in national statistics. Furthermore, an individual-level approach is also required. Among Korean SMW in our study, bisexual women were vulnerable to poor mental health. We need to pay attention and create affirmative interventions for bisexual women. In addition, programs for those with low family support are necessary.

This study has limitations. First, since we recruited the study participants through LGBTQ organizations, it is possible that sexual minorities who were not affiliated with these groups may have been excluded. Second, to overcome the first limitation, we additionally recruited as many SMW as possible through social media. This may explain the relatively small portion of SMW over 40 years old in our study, which leads to the limitation of the generalizability for older SMW in Korea. Follow-up research, therefore, is needed at the national level and also for SMW over 40 years old. Third, there was no screening to assess whether our participants had been diagnosed with mental illness or were taking drugs, including anti-depressants. Finally, since our study is cross-sectional, it is difficult to completely infer a causality among variables even though the correlations between them were significant. Despite these limitations, we believe that this study is valuable given the findings above.

## 5. Conclusions

The mental HRQoL of Korean SMW in this study was low, and their physical HRQoL was average. Korean SMW with high minority stress or low social support showed low mental HRQoL. Additionally, Korean bisexual women had lower mental HRQoL than lesbians. In terms of the mental health of Korean SMW, it is necessary to lower minority stress and increase social support. Particular attention is needed for bisexual women. Moreover, since the level of physical activity had a significant relationship with physical HRQoL, it is necessary to develop a comprehensive health promotion program for the Korean SMW.

## Figures and Tables

**Table 3:** Predictors on Physical Health-related Quality of Life (*N* = 736).

Variables		Model 1				Model 2				Model 3				Model 4			
		B	β	t	*p*	B	β	t	*p*	B	β	t	*p*	B	β	t	*p*
(Constants)		52.00		72.61	<0.0001	53.22		61.93	<0.0001	57.55		37.00	<0.0001	54.17		25.42	<0.0001
Age	19–29(ref)																
	30–39	−0.52	−0.03	−0.65	0.52	−0.24	−0.01	−0.30	0.77	−0.51	−0.03	−0.64	0.52	−0.60	−0.03	−0.75	0.45
	40–51	0.19	0.00	0.13	0.90	0.64	0.02	0.44	0.66	−0.03	0.00	−0.02	0.98	−0.37	−0.01	−0.25	0.80
City	Capital city	0.37	0.02	0.66	0.51	0.42	0.03	0.74	0.46	0.42	0.03	0.73	0.46	0.45	0.03	0.79	0.43
Education	Above College	−1.81	−0.07	−1.84	0.07	−1.61	−0.06	−1.65	0.10	−1.65	−0.06	−1.70	0.09	−1.35	−0.05	−1.38	0.17
Income	≥$50,000	1.01	0.06	1.63	0.10	0.81	0.05	1.32	0.19	0.89	0.05	1.45	0.15	0.76	0.04	1.24	0.22
Employment	Employed(ref)																
	Student	1.64	0.11	2.39	0.02	1.47	0.10	2.16	0.03	1.47	0.10	2.17	0.03	1.43	0.09	2.12	0.03
	Unemployed	1.71	0.06	1.60	0.11	1.80	0.07	1.69	0.09	1.85	0.07	1.75	0.08	1.97	0.07	1.86	0.06
Sexual Identity	Bisexual	−1.56	−0.10	−2.69	0.01	−1.72	−0.11	−2.98	<0.001	−1.92	−0.13	−3.34	<0.001	−1.93	−0.13	−3.36	<0.001
Smoking	Yes					−1.16	−0.06	−1.70	0.09	−1.12	−0.06	−1.66	0.10	−1.12	−0.06	−1.66	0.10
High-risk Drinking	Yes					−0.55	−0.02	−0.62	0.54	−0.60	−0.02	−0.68	0.50	−0.69	−0.03	−0.78	0.43
Physical Activity	High(ref)																
	Medium					−0.31	−0.02	−0.48	0.63	−0.26	−0.02	−0.41	0.68	−0.19	−0.01	−0.29	0.77
	Low					−2.70	−0.15	−3.67	<0.001	−2.69	−0.15	−3.68	<0.001	−2.48	−0.14	−3.38	<0.001
Minority Stress										−1.96	−0.12	−3.33	<0.001	−1.76	−0.11	−2.98	0.003
Perceived Social Support														0.60	0.09	2.32	0.02
R^2^		0.032				0.057				0.072				0.079			
Adjusted R^2^		0.021				0.042				0.055				0.061			
F		2.999				3.671				4.291				4.392			
*p*		0.003				<0.0001				<0.0001				<0.0001			

ref. = reference. Model 1: Age group, City, Education level, Annual family income, Employment status, Sexual identity. Model 2: Age group, City, Education level, Annual family income, Employment status, Sexual identity, Smoking, Physical activity. Model 3: Age group, City, Education level, Annual family income, Employment status, Sexual identity, Smoking, Physical activity, Minority stress. Model 4: Age group, City, Education level, Annual family income, Employment status, Sexual identity, Smoking, Physical activity, Minority stress, Perceived social support.

**Table 4:** Predictors on Mental Health-related Quality of Life (*N* = 736).

Variables		Model 1				Model 2				Model 3				Model 4			
		B	β	t	*p*	B	β	t	*p*	B	β	t	*p*	B	β	t	*p*
(Constants)		46.66		34.93	<0.0001	42.54		230.25	<0.0001	56.38		22.64	<0.0001	31.704		10.06	<0.0001
Age	19–29(ref)																
	30–39	0.37	0.01	0.28	0.78	0.69	0.02	0.53	0.60	−0.19	−0.01	−0.15	0.88	−0.82	−0.03	−0.69	0.49
	40–51	4.93	0.08	2.06	0.04	5.36	0.09	2.25	0.03	3.21	0.05	1.37	0.17	0.73	0.01	0.34	0.74
City	Capital city	0.57	0.02	0.61	0.54	0.51	0.02	0.55	0.58	0.49	0.02	0.54	0.59	0.72	0.03	0.87	0.39
Education	Above College	−3.02	−0.07	−1.89	0.06	−2.83	−0.07	−1.77	0.08	−2.95	−0.07	−1.90	0.06	−0.73	−0.02	−0.50	0.62
Income	≥$50,000	1.95	0.07	1.93	0.05	1.72	0.06	1.71	0.09	1.96	0.07	2.00	0.05	1.03	0.04	1.14	0.26
Employment	Employed(ref)																
	Student	−3.23	−0.13	−2.89	<0.0001	−3.36	−0.13	−3.02	<0.001	−3.37	−0.13	−3.11	<0.001	−3.63	−0.14	−3.64	<0.0001
	Unemployed	−6.94	−0.15	−3.99	<0.0001	−7.06	−0.16	−4.04	<0.0001	−6.88	−0.15	−4.05	<0.001	−6.05	−0.13	−3.87	<0.0001
Sexual Identity	Bisexual	−2.16	−0.09	−2.29	0.02	−2.33	−0.09	−2.47	0.01	−2.99	−0.12	−3.24	<0.001	−3.04	−0.12	−3.57	<0.0001
Smoking	Yes					−2.01	−0.07	−1.80	0.07	−1.89	−0.06	−1.74	0.08	−1.87	−0.06	−1.87	0.06
High−risk Drinking	Yes					0.83	0.02	0.57	0.57	0.66	0.02	0.47	0.64	0.02	0.00	0.01	0.99
Physical Activity	High(ref)																
	Medium					−1.52	−0.06	−1.44	0.15	−1.37	−0.05	−1.34	0.18	−0.82	−0.03	−0.87	0.38
	Low					−2.94	−0.10	−2.44	0.02	−2.90	−0.10	−2.48	0.01	−1.38	−0.05	−1.27	0.20
Minority Stress										−6.24	−0.24	−6.65	<0.0001	−4.81	−0.18	−5.50	<0.0001
Perceived Social Support														4.35	0.38	11.41	<0.0001
R^2^		0.063				0.075				0.128				0.262			
Adjusted R^2^		0.053				0.060				0.113				0.250			
F		6.147				4.889				8.180				18.262			
*p*		<0.0001				<0.0001				<0.0001				<0.0001			

ref. = reference. Model 1: Age group, City, Education level, Annual family income, Employment status, Sexual identity Model 2: Age group, City, Education level, Annual family income, Employment status, Sexual identity, Smoking, Physical Activity model 3: Age group, City, Education level, Annual family income, Employment status, Sexual identity, Smoking, Physical activity, Minority stress Model 4: Age group, City, Education level, Annual family income, Employment status, Sexual identity, Smoking, Physical activity, Minority stress, Perceived social support.

## Data Availability

The data are not publicly available due to privacy or ethical restrictions.
